# Salinity strengthens plant–pollinator mutualism through leaf physiological status in grafted tomatoes

**DOI:** 10.1007/s00425-026-05110-y

**Published:** 2026-08-18

**Authors:** Ángela S. Prudencio, Cristina Martínez-Andújar, José Ángel Martín-Rodríguez, Rim Ben Youssef, Purificación A. Martínez-Melgarejo, Juan José Guerrero, Maialen Ormazabal, Francisco Pérez-Alfocea

**Affiliations:** 1 Department of Plant Nutrition, Centro de Edafología y Biología Aplicada del Segura (CEBAS-CSIC), Campus Universitario de Espinardo, 25, 30100 Murcia, Spain; 2 Laboratory of Extremophile Plants, Biotechnology Center of Borj-Cedria, P.O. Box 901,, 2050 Hammam-Lif, Tunisia

**Keywords:** Agrosystemic adaptation, *Bombus terrestris*, Pollinator-assisted phenotyping, Rootstock breeding

## Abstract

**Main conclusion:**

The interaction between rootstock-mediated leaf Na and nutrient concentrations, and the subsequent impact on *Fv/Fm* and unbalanced C/N are major traits associated with plant growth, yield, and bumblebees’ pollinating activity under salinity.

**Abstract:**

Soil and water salinization compromises crop yield by reducing plant growth and reproductive fitness through altering source-sink activities, which in turn, affects pollination ecosystem services. However, while pollination services are barely considered as crop breeding targets, little is known about how stress-induced changes in plants affect pollinators’ nutritional landscape and their foraging decisions. To address this gap, visually assessed pollination activity of managed bumblebees (*Bombus terrestris*) was monitored in a tomato commercial cultivar (*Solanum lycopersicum* L. Mill) grafted onto 26 experimental rootstocks, and related to agronomic and leaf/flower physiological traits under optimal (control) and saline (75 mM NaCl) conditions. Rootstock and salinity generated variability in agronomic (biomass, fruit yield and quality) and physiological (leaf and flower ionomics, chlorophyll fluorescence) traits, as well as in pollinator foraging behavior (flowers visited and preference). Based on a correlation analysis, the environmental pressure imposed by salinity strengthened plant–pollinator mutualistic interactions since rootstock-mediated fruit yield and plant vigor were associated not only with the leaf nutrient (C, N, P, K, Ca, S, Zn) and *Fv/Fm* status, but also with bumblebees’ activity. Leaf Na concentration emerged as the strongest negative factor associated with plant growth, physiology and yield as well as with pollinator activity, while flower abortion was more closely linked to leaf C, N and P status rather than Na levels in flowers. The results suggest that pollinator foraging decisions under salinity may reflect the plant physiological status and C/N balance, supporting a potential role for pollinators as ecosystem phenotypers for plant selection and breeding under environmental pressure.

**Graphical abstract:**

**Figure:**
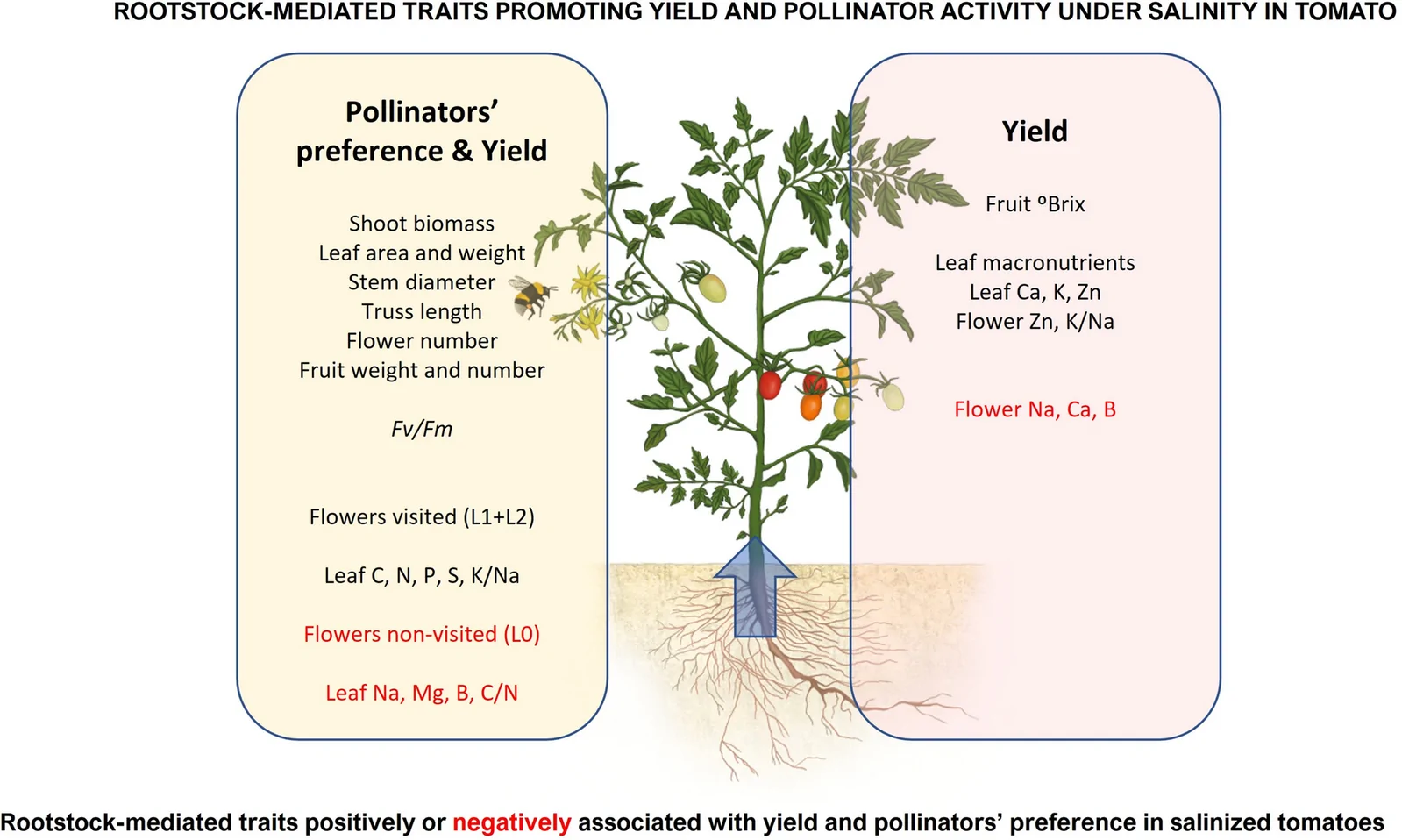

**Supplementary Information:**

The online version contains supplementary material available at https://doi.org/10.1007/s00425-026-05110-y.

## Introduction

Salinity in water and soil, aggravated by high temperatures, compromises crop yield of horticultural species by impacting the physiology of the plant and beneficial ecosystem services, such as insect pollination (Garibaldi et al. 2011; Potts et al. 2016; Liang et al. 2024). One of the strategies adopted by breeders and farmers is the selection of the most efficient varieties to cope with salt stress. Hence, plants can retain crop productivity under salinity by avoiding excessive stomatal closure and Na accumulation in leaves, and maintaining photosynthesis and meristematic activity through the production and transport of photoassimilates from source leaves to vegetative and reproductive sink tissues, as flowers and fruits (Pérez-Alfocea et al. 2010; Albacete et al. 2014; Liang et al. 2024). Root performance is determinant to ensure water and nutrient uptake but also contributes to the ionic and osmotic balance maintaining a functional physiological status in the shoot (Moles et al. 2016). For instance, accumulation of saline ions in the root prevents their translocation to the shoot and leaves (Pérez-Alfocea et al. 1993) and ultimately the maintenance of Na/K ratio homeostasis (Romero-Aranda et al. 2020). Moreover, the root-to-shoot hormonal regulation of carbon metabolism is central to manage saline stress for keeping optimum source-sink relationships and avoiding photosynthetic feedback inhibition (Albacete et al. 2014; Munns et al. 2020; Martínez-Andújar et al. 2021). Therefore, grafting a commercial variety onto experimental rootstocks is a powerful tool to explore the genetic variability and select the best rootstock × scion combinations enabling a suitable balance between stress adaptation and crop stability (Santa-Cruz et al. 2002; Albacete et al. 2008, 2015; Martínez-Andújar et al. 2020; Niu et al. 2022).

Salt tolerance in tomato (*Solanum* sp.) is still present in wild species while it has been lost during domestication (Héreil et al. 2024; Wang et al. 2024). However, the direct transfer of tolerant traits from wild rootstocks to elite varieties is possible (Albacete et al. 2009; Asins et al. 2015). Besides the constitutive genetic capacity, salinity induces morphological and physiological changes that affect root development and shoot physiology through root-to-shoot hormonal communication (Cuartero and Fernández-Muñoz 1998; Munns et al. 2020). Flower fertilization is also negatively affected by salinity, mainly due to altered source-sink relationships rather than toxic ion accumulation, since Na is allocated in flower organs other than pollen, although pollen production can be reduced (Ghanem et al. 2009) as well as the number of flowers (Grunberg et al. 1995). Compared to leaves, the effects of salinity on flower composition have been little explored despite their contribution to flower fertility and interaction with animals that contribute to pollination and fruit set. Ultimately, flower nutrient composition for pollinators would depend on interactions taking place in the rhizosphere and the ability of the rootstock to catch ions and water needed for metabolite synthesis under each environment (Jones and Rader 2022; Dorey and Schiestl 2024).

Since artificial (hand-operated vibrators) or natural (managed pollinators as bumblebees, *Bombus terrestris*, used in this study) induced pollination is common in greenhouse tomatoes to maximize fruit set particularly under suboptimal environmental conditions like low temperature, the relationship between salinity and pollinators is becoming an emerging topic to be studied (Lovett and Carr 2023; Marroquin et al. 2023). Artificial sodium-enriched nectar applied to flowers from different species was more visited by pollinators (Finkelstein et al. 2022; Lovett and Carr 2023). On the other hand, nutrients such K or P can reach repelling concentrations for pollinators in avocado nectar (Afik et al. 2014). Such phenomenon has not been checked in anthers or pollen. As plants, pollinators need to keep a balance of nutritionally important elements other than Na, such as macronutrients P, K, S, Ca, and Mg and micronutrients Fe and Zn (Lau et al. 2023). Moreover, some elements such as Na, N, P, K, S and Zn can be limiting in pollen (Filipiak et al. 2017). Therefore, mineral elements in flowers are important for both fertility and pollinators’ wellness, and the pollination service that they provide.

Since the mutualistic relations depend on pollinator health and crop pollination gains it is important to understand how both (insect and plant) nutritional demands interact with crop environment (Jones and Rader 2022). Indeed, the effects of abiotic stresses on plant–pollinator interactions ensure pollination services in agroecosystems, as agriculture is one of the driving forces for population dynamics of pollinating insects (Krama et al. 2022; Murphy et al. 2022). Several studies have addressed the effects of soil mineral nutrition on pollinator visitation, mainly managing N, P and or K elements (Muñoz et al. 2005; Vaudo et al. 2022; Martínez-Andújar et al. 2023). By using a conventional observational method (Martínez-Andújar et al. 2023) or an RFID-based system (Ormazabal et al. 2024; Jiménez et al. 2026) to monitor pollination activity, it was reported that bumblebees were sensitive to grafting of a commercial variety onto different rootstocks. Moreover, the environmental pressure imposed by nutrient (NPK) deficiency strengthened the preference of bumblebees for plants with improved leaf nutritional status mediated by the rootstocks, and its positive effect on photosynthesis and yield (Martínez-Andújar et al. 2023). Such results support the use of plant–pollinator mutualistic interactions as a selection tool for increasing yield and yield stability under any environmental pressure (Pérez-Alfocea et al. 2024).

The novelty of this study resides in evaluating how rootstock-mediated *Solanum* sp. biodiversity influences the mutualistic interaction between a tomato variety in interaction with the environmental pressure in the rootzone (salinity) and the ecosystem pollinating service provided by bumblebees through changes in leaf and flower physiological and nutritional status. Unlike previous work under macronutrient (NPK) deficiency, salinity imposes both osmotic and ionic constraints, particularly through Na accumulation, which may differentially affect plant performance and its interaction with pollinators. For this purpose, we analyzed the visually assessed pollinators’ activity in relation to plant growth, yield components, leaf and flower mineral composition, chlorophyll fluorescence, pollen viability and fruit quality under control and salinity conditions. The alignment of bumblebees’ pollination activity (as a proxy of foraging preference) with rootstock-mediated impact on productivity and plant adaptation to salinity in a unique commercial variety is discussed.

## Material and methods

### Plant material, grafting, and growth conditions

The cherry tomato *Solanum lycopersicum* cv. ‘Unidarkwin’ (Unigenia Semillas SLU, Los Alcázares, Spain) was grafted onto 26 experimental rootstocks. Moreover, non-grafted (NG, denoted as rootstock #1 in the figures) and self-grafted (SG, #2) plants of the scion variety, as well as plants grafted onto the widely used commercial F1 rootstock ‘Maxifort’ (#3) (Bayer–De Ruiter, Naaldwijk, Netherlands), were used as internal controls and as an external reference, respectively. The experimental rootstocks (#4–20) derived from crosses between *S. lycopersicum* cv. M82, *S. pimpinellifolium* LA1589 and *S. lycopersicum* cv. TA209 with introgression lines (*S. lycopersicum* cv. M82 x *S. pennellii* LA0716 and *S. lycopersicum* E6203 x *S. habrochaites* LA1777) and *S. lycopersicum* cv. TLO1749. In addition, three hormonal mutant lines affected in abscisic acid (#22) and ethylene (#24, 25) production, some of their F1 crosses (#26–29), and their WT backgrounds Ailsa Craig (#21) and MicroTom (#23), respectively, were also tested as rootstocks. ABA and ethylene are involved in rootstock-mediated responses to salinity and nutrient stress in tomato (Albacete et al. 2009; Martínez-Andújar et al. 2016, 2017, 2021).

The sowing of scion and rootstock seeds was synchronized to obtain the appropriate stem diameter to ensure grafting viability and homogeneous grafted plants, as described in Martínez-Andújar et al. (2023). Forty days after grafting, 12 plants per graft combination were transferred to a commercial-like plastic greenhouse, randomly distributed in two blocks (6 plants per genotype), and cultivated in sandy soil until completing the growing cycle. All plants were initially irrigated with complete Hoagland’s solution (Hoagland and Arnon 1950) for 30 days. Then, half of the plants received 75 mM NaCl added to the nutrient solution until the end of the experiment (100 days under salinity). The macro (in mM) and micronutrient (in µM) concentrations in the nutrient solutions were as follows: N, 12; P, 2; K, 6; Ca, 3.5; Mg, 1; S, 1; Fe, 100; B, 22.64; Mn, 3.14; Zn, 2.09; Cu, 0.60; Mo, 0.06. The average electrical conductivities (EC) of the fertigation solutions were 4 dS m^−1^ (control treatment) and 8.9 dS m^−1^ (saline treatment). Plants received conventional phytosanitary treatments compatible with the commercial use of managed bumblebees.

### Fruit yield, quality and vegetative growth parameters

Fruit yield per plant was weekly until the end of the experiment (100 days under salinity). Fruit number and weight, fruit truss length and weight, flower abortion number and abortion index were also scored. Leaf area and leaf fresh weight were determined in the same mature leaves used for chlorophyll and nutritional status. Plant vegetative growth parameters as stem diameter and shoot and root fresh weights were also measured at the end of the experiment. Soluble Solids Content (SSC) in ripe fruits (°Brix) was measured using a digital refractometer Atago 3810 (ATAGO Co., Ltd., Tokyo, Japan).

### Chlorophyll fluorescence, foliar and flower nutritional analysis

Chlorophyll status was assessed in the leaves just below the 7th truss after 70 days of salt treatment in 2–6 plants from each genotype × treatment combination. The maximum quantum yield of open photosystem II (PSII) (*Fv/Fm*) was calculated according to previous literature (Maxwell and Johnson 2000), as described in Albacete et al. (2009), using a FluorPen FP100 (Photon Systems Instruments, Drasov, Czech Republic). After 95 days of starting the salt treatment, samples from the same leaves (or equivalent) described above and flowers were collected for nutritional status assessment. Plant material was dried for 48 h at 80 °C, milled to a powder, and digested with a HNO_3_:HClO (2:1, v/v) solution. Macronutrients (Ca, K, Mg, Na, P, S) and Micronutrients (Mn, Fe, B and Zn) were analyzed in 50 mg (flower) or 200 mg (leaf) of dry tissue by inductively coupled plasma spectrometry (ICP-OES IRIS INTREPID II XDL, Thermo Fisher Scientific Inc., Loughborough, UK) available at CEBAS-CSIC analytical services (https://www.cebas.csic.es/servicios/serv_ionomica.html). Total C and N concentrations were determined in the same samples by the combustion method using a LECO TRUSPEC analyzer (Geleen, Netherlands) by the same service.

### Pollinator preference quantification

Pollinator activity was monitored following an indirect observational method as described in Martínez-Andújar et al. (2023). Managed hives (Agrobio S.L., Almería, Spain) of pollinating insects (*Bombus terrestris*) were placed following commercial guidelines to ensure pollination of all the plants in the greenhouse. Pollinators’ foraging preferences were evaluated weekly throughout the complete reproductive stage of the plant, for which the flower trusses already assessed were labeled. Quantification of pollinators’ preferences was proxied by scoring the brown marks that the bites of the insects leave on the stamens during their visits to the flowers, so that Level 0 (L0), Level 1 (L1), and Level 2 (L2) correspond to absence, low intensity and high intensity of bites (as a percentage with respect to the total flowers assessed across the whole period), respectively. The percentage of flowers visited was calculated as L1 + L2, while L1 and L2 can be used as proxies of short and long visits, accordingly. On average, six flowering trusses per plant were assessed (17–18 flowers). At least three replicates (plants) per genotype and treatment were monitored by four different observers previously trained according to the scoring criteria described by Martínez-Andújar et al. (2023), which makes a total of 3448 flowers assessed in 200 plants.

### Pollen viability

The impact of rootstock-treatment effect on pollinator activity through pollen viability was assessed. Pollen was collected in the afternoon from fully opened flowers not visited by pollinators by shaking the anthers using an electric shaker. Samples were stored in 1.5-mL Eppendorf tubes at 4 °C until analysis. Pollen grains were stained with a carmine acetate solution in porta preparations to evaluate the presence of cytoplasm (Vassileva et al. 1991), and distinguish between viable and non-viable grains under a stereoscopic microscope Olympus SZ61 (Olympus, Hamburg, Germany). The percentage of pollen viability was calculated in sets of at least 300 pollen grains in 2–4 replicates per genotype-treatment combination.

### Statistical analysis

Data analysis was carried out in R programming environment (R-4.5.3 for Windows; https://www.r-project.org) and Microsoft Excel (Microsoft Corporation, Redmond, Washington, USA). Analysis of variance (ANOVA) test was used to detect significant differences: one-way ANOVA was applied to compare genotypes within each treatment (using “aov” function), and type III ANOVA was used to evaluate the effects of factors genotype, treatment, and their interaction (using the “Anova” function from car package). Tukey’s post-hoc test was performed using the “TukeyHSD” function and multcompview package was used for visualization of significant differences with letters between groups. Homogeneity of variances was assessed using Levene’s test (“leveneTest” function from the car package), while normality of residuals was evaluated using the Shapiro–Wilk test (“shapiro.test”), along with visual inspection of histograms and Q-Q plots. When assumptions of normality and homoscedasticity were not met, nonparametric approaches were applied. For one-way comparisons, the Kruskal–Wallis test (“kruskal.test” function) was applied. For factorial designs, the Aligned Rank Transform (ART) ANOVA was used via the ARTool package (“art” function), allowing testing of main effects and interactions. Correlation analysis were performed using the Hmisc package; however, given the elevated number of multiple pairwise comparisons, the possibility of false-positive correlations cannot be excluded. Figures were built using the pheatmap package for heatmaps, and ggplot2 package or Microsoft Excel for other plots. Figures drawn with R and Microsoft Excel were edited using Inkscape® software (https://inkscape.org/).

## Results

### Fruit yield and related agronomic and physiological traits

**Fig. 1:**
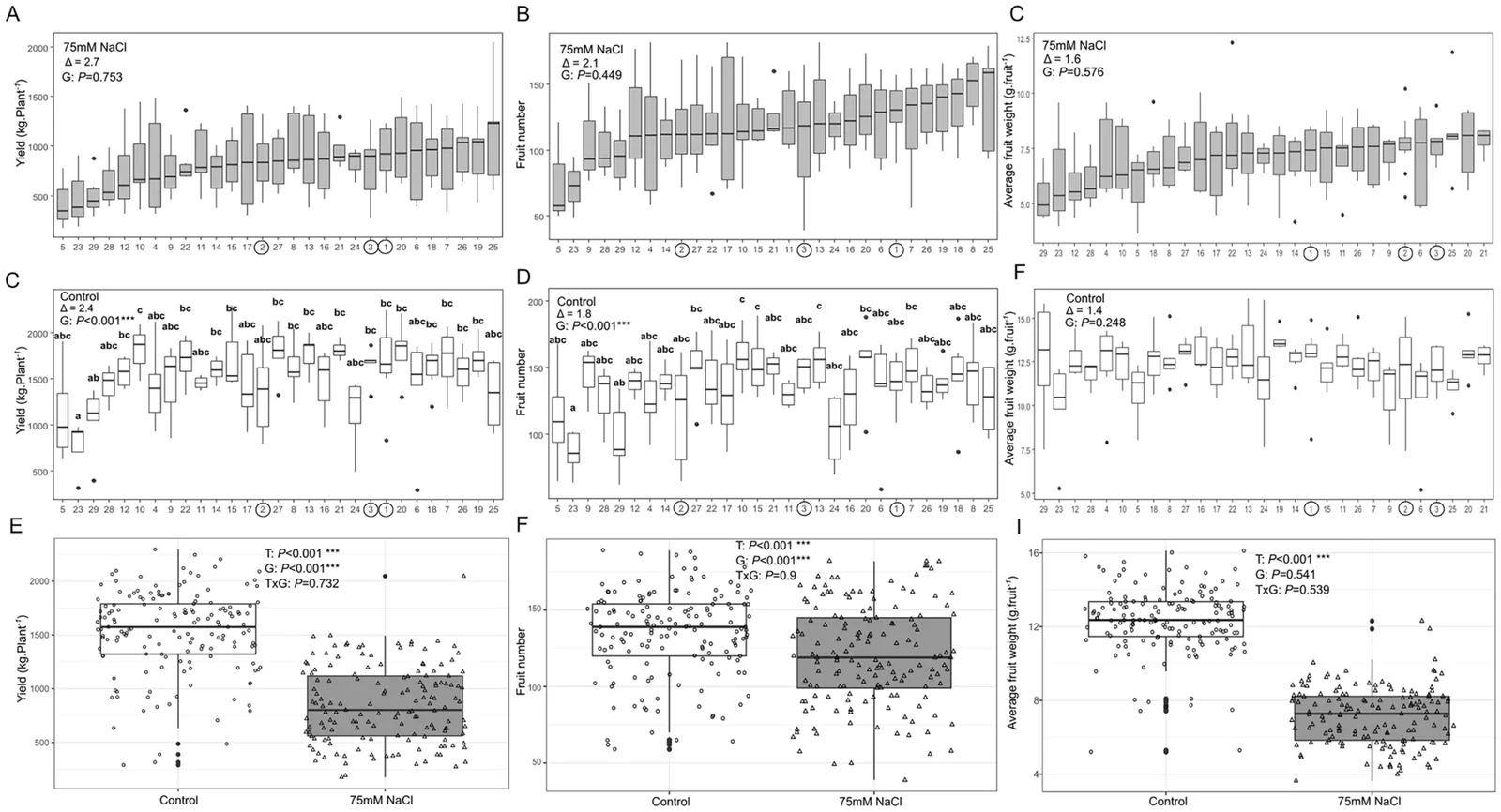
**A**–**F** Boxplot showing the yield, fruit number and average fruit weight of *Solanum lycopersicum* cv. ‘Unidarkwin’ grafted onto different rootstocks growing under salinity (**A**–**C**) and control (**D**–**F**) conditions during 100 days. *n* = 3–6, except for non-grafted under control (circled #1, *n* = 7) and for the self-grafted under control (circled #2, *n* = 9) and NaCl (*n* = 11) conditions. Commercial rootstock ‘Maxifort’ corresponds to genotype number #3 (circled). Letters indicate significant differences according to Tukey’s test. **G**–**I** Boxplot with individual data points showing the yield and fruit number by the treatment (*n* = 157 for control, 153 for NaCl). Treatment is indicated both by boxplot color (white for control and grey for NaCl) and point shape (circles for control and triangles for NaCl). Fold change between the highest and the lowest yield rootstock is indicated in the top left of the panel. *P*-values for G within T (ANOVA/Kruskal–Wallis) and for factorial effects (T, G, T × G) indicated. T, treatment; G, genotype; T x G, their interaction (in all legends)

**Fig. 2:**
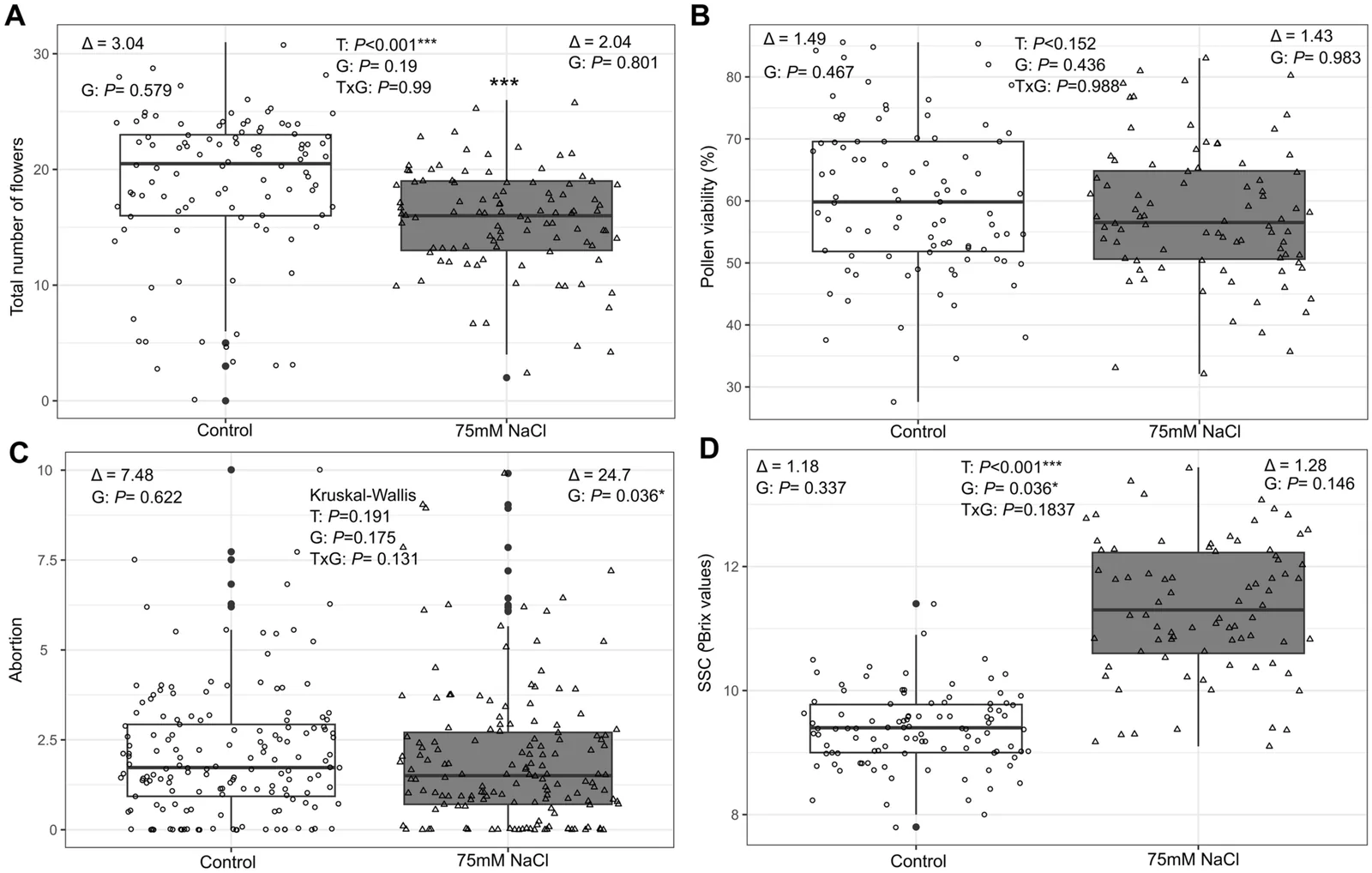
**A**–**D** Boxplot with individual data points showing the number of flowers (**A**, *n* = 102 for control and *n* = 98 for NaCl conditions), percentage of pollen viability (**B**, *n* = 91 for control and *n* = 84 for NaCl conditions), flower abortion (**C**, *n* = 157 for control and *n* = 153 for NaCl conditions) and °Brix values (**D**, *n* = 104 for control and *n* = 84 for NaCl conditions) of *Solanum lycopersicum* cv. ‘Unidarkwin’ plants grafted onto 29 different rootstocks during 100 days of treatment. Δ indicates fold change in each case between the highest and lowest rootstock, on average. *P*-values for G within T (ANOVA/Kruskal–Wallis) and for factorial effects (T, G, T × G) indicated

Experimental rootstocks induced 2.7, 2.1, and 1.6-fold variation in fruit yield, fruit number and fruit weight of the commercial variety under NaCl treatment, respectively (Fig. 1A–C). Under control conditions, these rootstock-induced variations were 2.4, 1.8, and 1.4, respectively (Fig. 1D–F). On average, salinity reduced fruit yield by 55.4%, which was mainly due to a reduction in fruit weight (42%) rather than in fruit number (12%), compared to control conditions (Fig. 1G–I). Moreover, salinity reduced the number of flowers by 20% (*P* < 0.001***), while it did not significantly affect pollen viability and flower abortion, compared to control conditions (Fig. 2A–C). Similarly, the average root biomass was unaffected by salinity (but significantly influenced by the rootstock genotype) (Fig. S1A). The lowered number of flowers under salinity was better explained by a 50–60% reduction of vegetative growth (shoot biomass, stem diameter, and leaf weight and area) (Fig. S1B–E). Leaf physiology status (assessed as *Fv/Fm*) was also negatively impacted by salinity (*P* < 0.001***) (Fig. S1F). The fruit quality, assessed as °Brix, increased by 15% under NaCl treatment (*P* < 0.001***), compared to control, and differed between rootstocks, regardless of the treatment (*P* = 0.073, Fig. 2D).

### Pollinators’ activity and preference

**Fig. 3:**
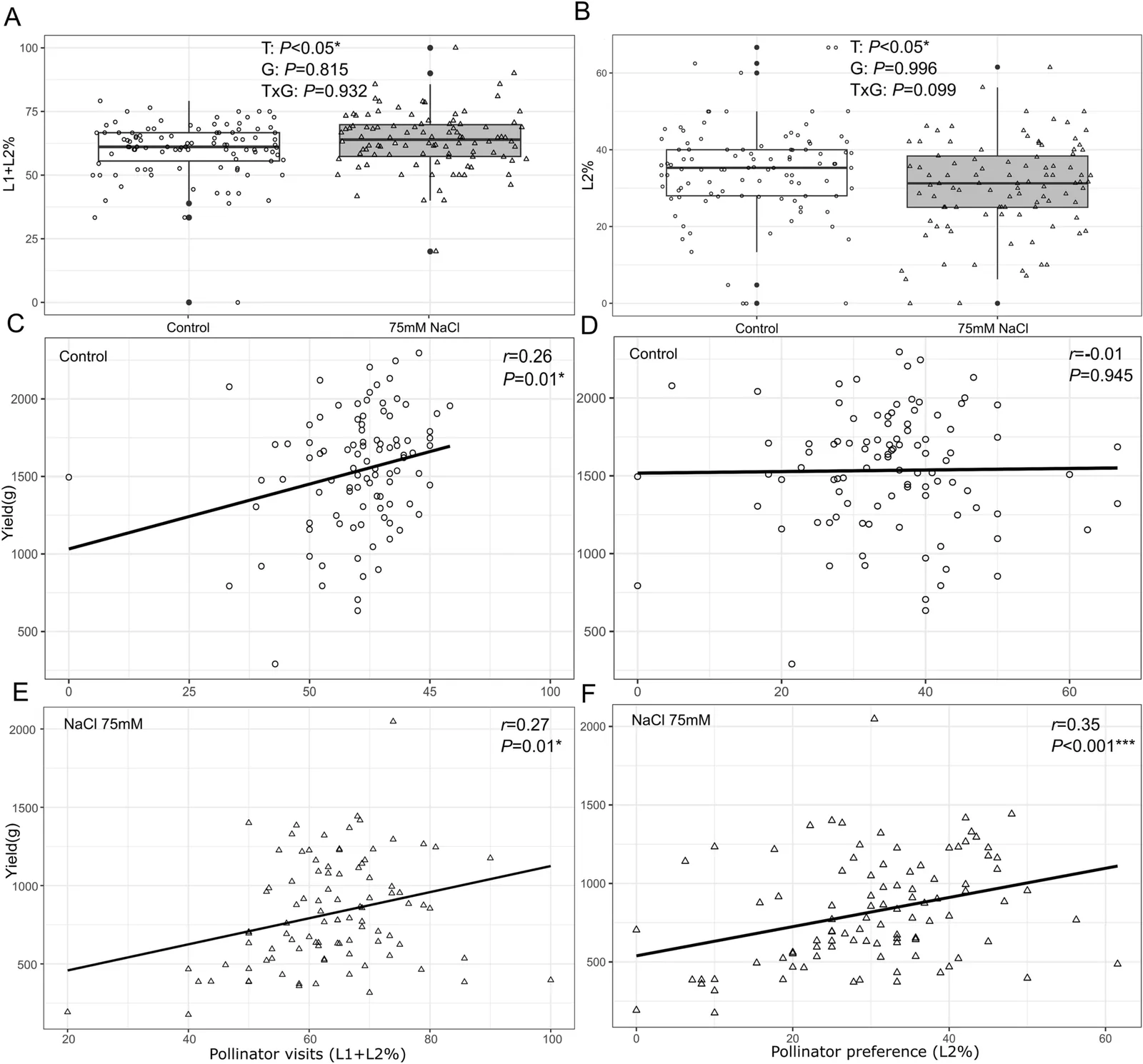
**A** Scatter plot with individual data points showing the percentage of flowers visited by bumblebees (L1 + L2) and **B** their preference expressed as the percentage of long visits (L2) under control (*n* = 101) and saline (*n* = 98) conditions (see Pollinator preference quantification in Material and methods). Significant effects of treatment (T), genotype (G), and their interaction (T × G) are indicated by asterisks (* = *P* < 0.05 and *** = *P* < 0.01) according to ANOVA test. **C**–**F** Correlation between pollinator’s visits (L1 + L2) or preference (L2) and total yield of *Solanum lycopersicum* cv. ‘Unidarkwin’ grafted onto different rootstocks growing under control, indicated by circles (*n* = 101) (**C**, **D**) and NaCl conditions indicated by triangles (**E**, **F**) during 100 days (*n* = 98). The values of Spearman correlation coefficient (*r*) and their *P* values are indicated

Based on the visual scoring system, salinity increased the average percentage of visited flowers (L1 + L2, Fig. 3A), and short visits (L1, Fig. S2A), but decreased the preference through long visits (L2, Fig. 3B) and the percentage of non-visited flowers (L0, Fig. S2B), compared to control conditions. Yield positively correlated with pollinator visits (L1 + L2), but not with pollination preference (L2), under control (Fig. 3C, D). However, under salinity, both pollination indicators positively and significantly correlated with fruit yield, mainly pollinators’ preference L2 (Fig. 3E-F). Moreover, the rootstocks induced a 1.5 (L1 + L2) to 1.7–1.8 (L2) fold variation in the pollinators’ activity under both growing conditions (Fig. S3).

### Leaf and flower nutritional status

#### Macro- and micro-nutrients in leaves and flowers

The salt treatment only reduced (12–13%) the total concentration of macro- (N, P, K, Mg, S and Ca) and micro-nutrients (Fe, Mn, B and Zn) in leaves but not in flowers, compared to control (Fig. S4A–B). In flowers, total macronutrients did not decrease significantly and even micronutrients increased (9%) under salinity conditions (Fig. S4C, D).

**Fig. 4:**
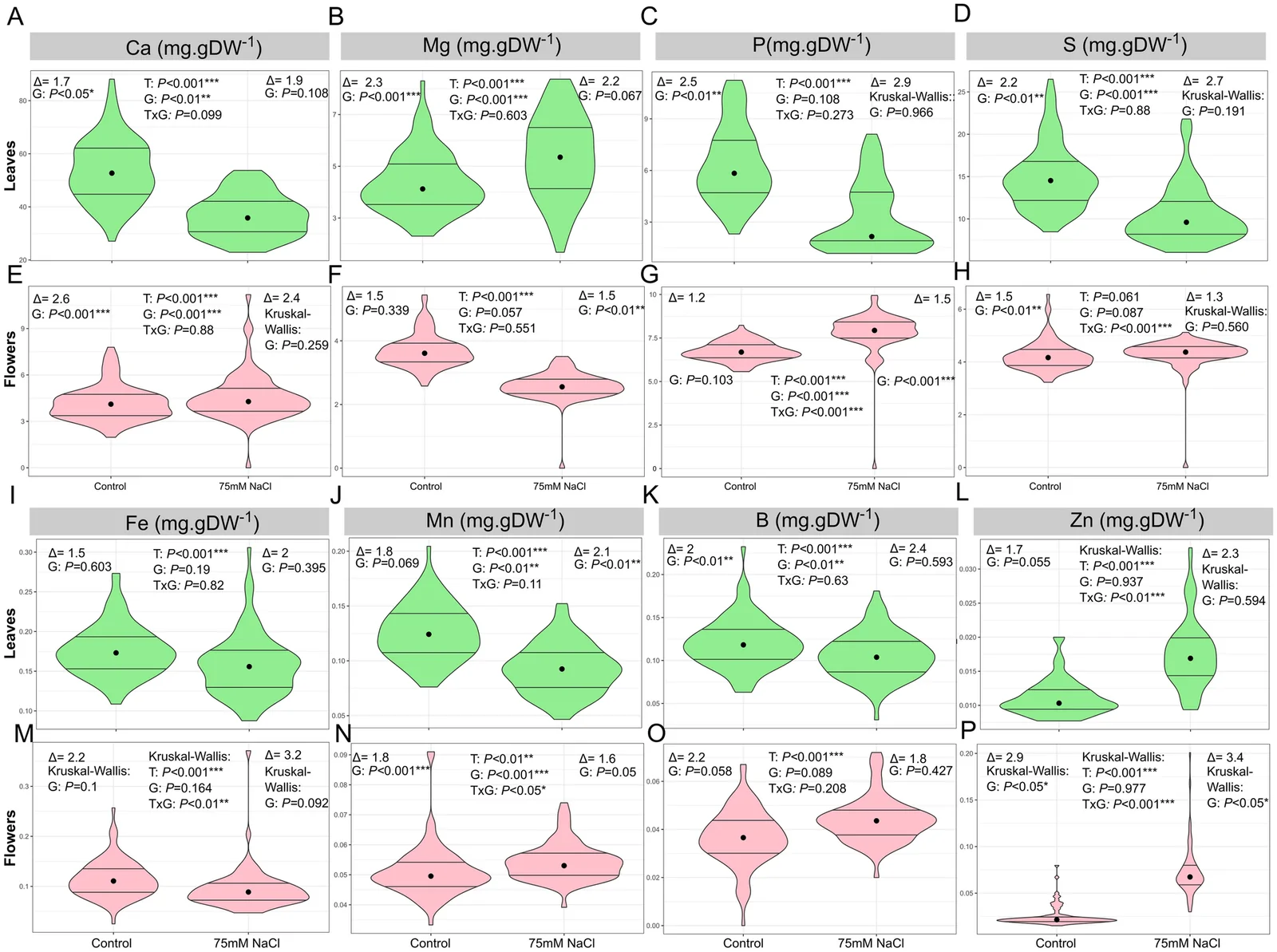
Violin plots showing concentrations of macronutrients (**A**–**H**) calcium, magnesium, phosphorus and sulfur (Ca, Mg, P and S) and micronutrients (**I**–**P**) iron, manganese, boron and zinc (Fe, Mn, B and Zn) in leaves (*n* = 86 for control and *n* = 85 for NaCl conditions) and flowers (*n* = 84–86 for control and *n* = 70–71 for NaCl conditions) from *Solanum lycopersicum* cv. ‘Unidarkwin’ plants grafted onto 29 different rootstocks grown under control and 75mM NaCl conditions during 95 days. Δ indicates fold change in each case between the highest and lowest rootstock, on average. *P*-values for G within T (ANOVA/Kruskal–Wallis) and for factorial effects (T, G, T × G) indicated

On average, salinity reduced most of macro (Ca, P, S) and microelements (Fe, Mn, and B) in the leaves in favor of the flowers, while the opposite was found for Mg, and an increase was observed for Zn in both organs (Fig. 4). Besides Na (see below), P registered the highest rootstock-mediated variability (Δ = 2.9) in salinized leaves (Fig. 4C). In flowers, the highest variability was observed for Ca (Δ = 2.4), Fe (Δ = 3.2) and Zn (Δ = 3.4) under salinity (Figs. 4E, M, P).

#### Total carbon and nitrogen in leaves and flowers

**Fig. 5:**
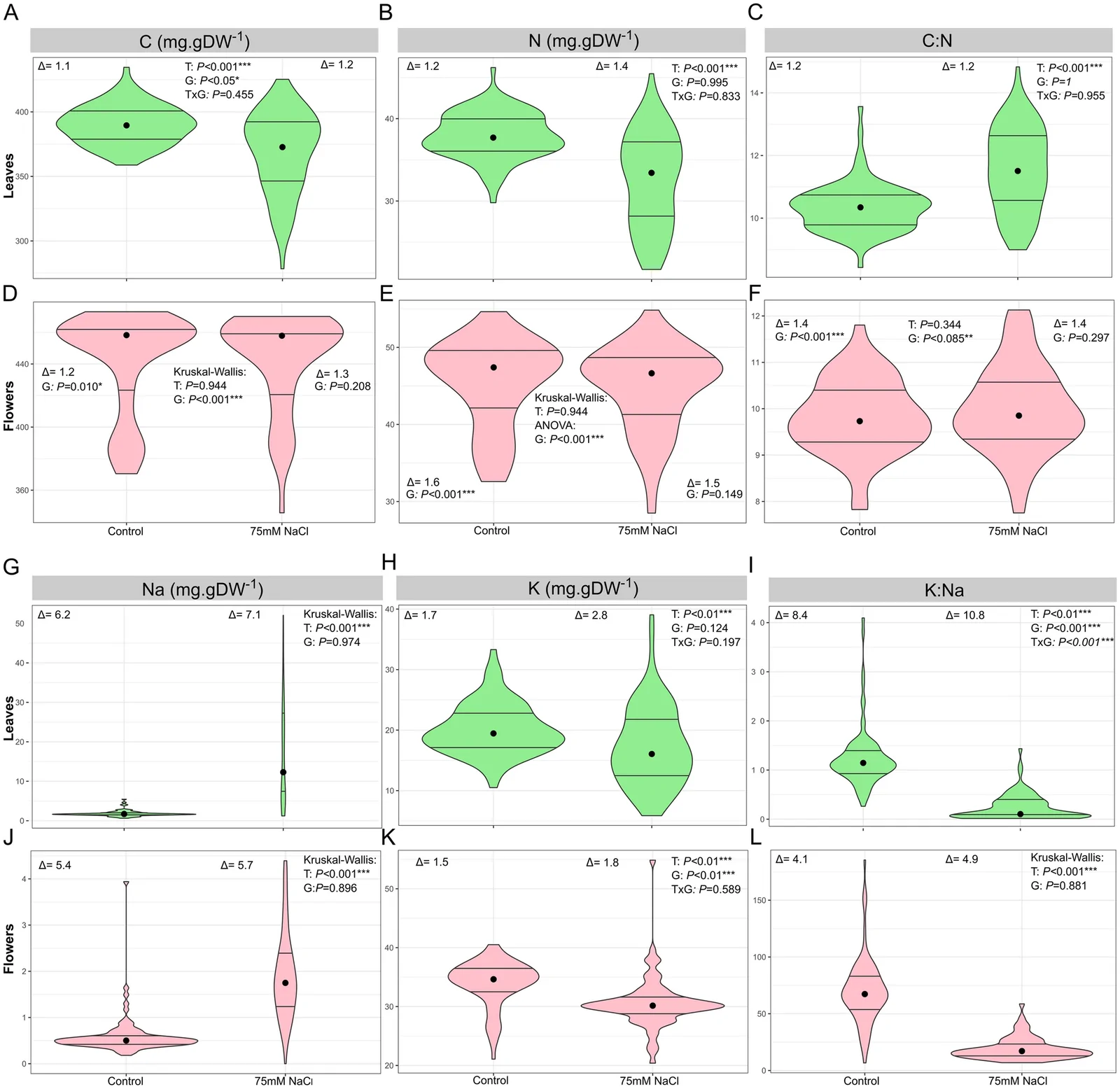
Violin plots showing ion C, N, Na, K concentrations, and C:N and K:Na ratios in *Solanum lycopersicum* cv. ‘Unidarkwin’ plants grafted onto different rootstocks growing under control and 75 mM NaCl conditions during 95 days. **A**–**F**: Concentrations of total carbon (C), nitrogen (N) and C:N index in leaves (*n* = 86 and 83) and flowers (*n* = 43 and 48). **G–L:** Sodium, potassium (Na, K) concentration and K:Na index in leaves (*n* = 86 and 85) and flowers (*n* = 86 and 72). The median is marked as a black point and the first and third quartiles are marked as a black line. Δ indicates fold change in each case between the highest and lowest rootstock graft, on average. *p* values for G within T (ANOVA/Kruskal–Wallis) and for factorial effects (T, G, T × G) indicated

The rootstock genotype significantly influenced leaf C but not N concentration under control conditions, while salinity decreased both elements (*P* < 0.001***) and increased the C/N ratio by 12% (*P* < 0.001***) compared with control conditions (Fig. 5A–C). In flowers, rootstock genotype but not salinity caused significant changes in these nutrient concentrations (Fig. 5D-F).

#### Sodium and potassium in leaves and flowers

Rootstock biodiversity provoked the highest variation in shoot Na concentration (Δ = 5.4–7.1) and K/Na ratio (Δ = 4.1–10.8) under control (Δ = 5.4–6.2) and saline (Δ = 5.7–7.1) conditions, being higher in salinized leaves (Δ = 7.1–10.8), and more stable in flowers (Δ = 5.7–4.9), compared to control conditions (Fig. 5G–L). Salinity provoked a 9- and fivefold increase in average Na concentration in leaves and flowers, respectively (Fig. 5G, J), along with a concomitant decrease in K concentration (Fig. 5H, K) and K/Na ratio (Fig. 5I, L), compared to control conditions. Interestingly, the rootstock-mediated variation in Na concentration in the scion was high even under control conditions, compared to K (Δ = 1.5–2.8 in both treatments), which was also reflected in the K/Na ratio. Those results suggest that the constitutive capacity of the rootstocks to regulate Na concentration in the scion is highly variable depending on the genetic background, and this trait could be highly relevant in the response to salinity of the chimeric plant.

#### Correlations between pollinator, physiological and yield related parameters

**Fig. 6:**
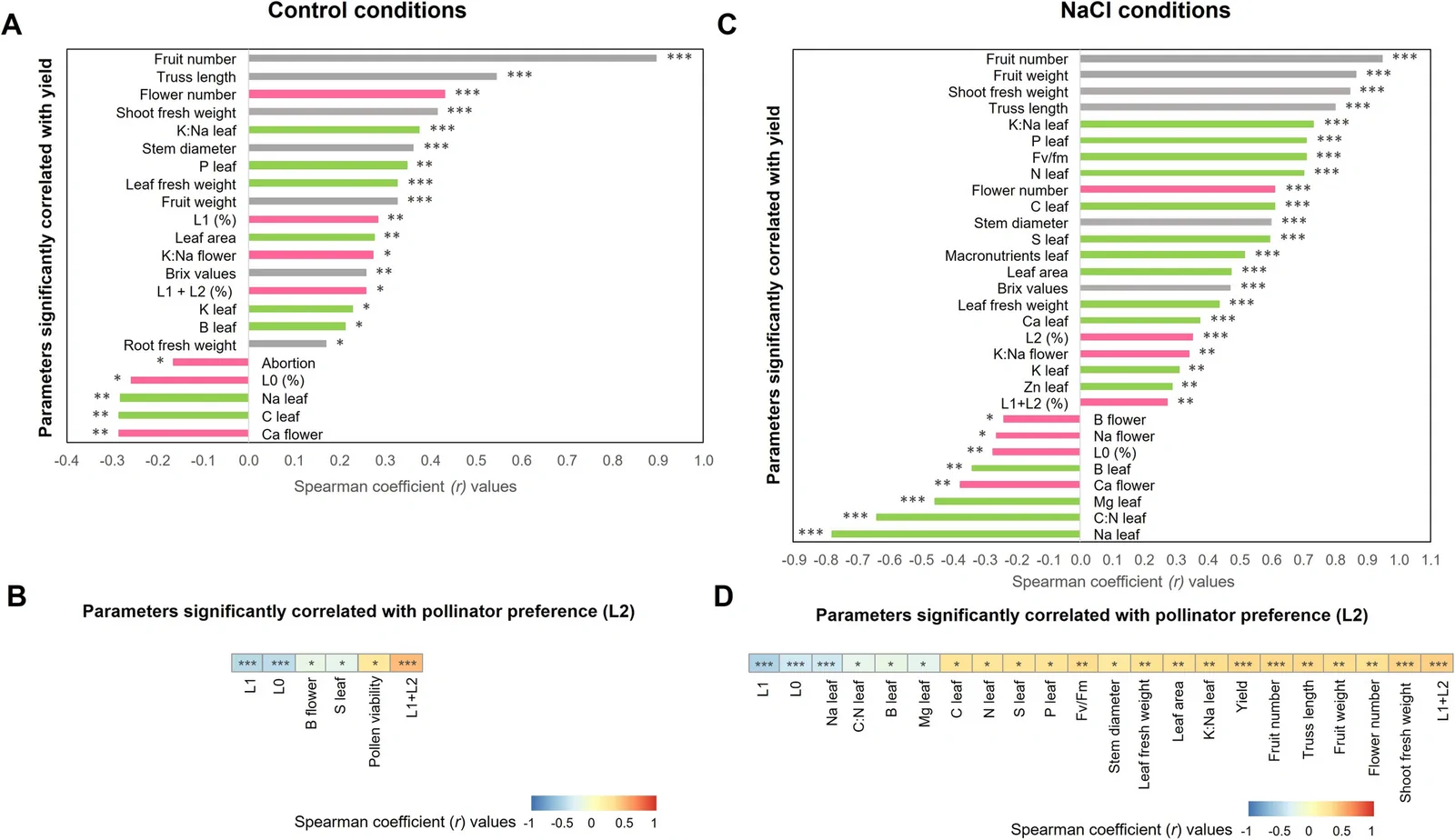
Parameters that significantly correlated with yield and pollinator preference (L2) under control (**A** and **B**) and NaCl (**C** and **D**) conditions. Spearman correlation coefficient is indicated by horizontal bars (**A**, **C**) or color legend (**B**, **D**) and level of significance is indicated by asterisks (*=*P*<0.05, **=*P*<0.01 and ***=*P*<0.001). In **A** and **C**, green (leaves) and pink (flowers) bars refer to nutrient/physiological traits, and gray bars refer to vegetative/reproductive parameters

Figure 6 shows the significant correlations among yield (A, C), bumblebees’ preference (B, D), the rest of agro-physiological parameters, nutrient concentrations, and pollination activity (L0, L1, L2, L1 + L2) under control (A, B) and NaCl (C, D) conditions. Figures S5 and S6 show complete heatmap correlations among all parameters under control and salt treatment conditions, respectively.

Under control conditions, fruit yield positively correlated with root fresh weight, number of flowers and fruits, leaf nutrients (P, K, B), K:Na ratio in both leaves and flowers, scion vegetative vigor, fruit °Brix values, and the percentage of flowers visited by bumblebees (L1 + L2), but not with the pollinator preference L2 (Figs. 6A, 3C–D). However, yield negatively correlated with C and Na in leaves, Ca in flowers, and flower abortion (Fig. 6A; S5). While bumblebees’ preference L2 under control conditions only correlated with pollen viability (Fig. 6B), flowers visited (L1 + L2) also correlated with other reproductive (truss length and fruit weight) and vegetative (leaf area and fresh weight, shoot fresh weight and stem diameter) parameters (Fig. S5). However, both preference (L2) and visits (L1 + L2) were negatively associated with B and S in flowers (Fig. 6B) and S in leaves (Fig. S5).

Under salinity, yield positively correlated with bumblebees’ visits and more significantly with their preference L2 (Figs. 6C, 3E–F). Moreover, rootstock-mediated variation in fruit yield positively correlated with fruit quality (°Brix), leaf nutritional status (C, N, P, K, Ca, S, Zn) and the maximum quantum efficiency of PSII (*Fv/Fm*) (Fig. 6C), an indicator of CO_2_ fixation (Genty et al. 1989; Edwards and Baker 1993). However, Na and some nutrients in flowers (Ca and B) correlated negatively with yield (Fig. 6C) and vegetative growth (Fig. S6). Intriguingly, Ca in leaves was strongly associated with growth, yield and *Fv/Fm* under salinity, while this nutrient in flowers negatively correlated with vegetative and yield parameters (Figs. 6C; S6).

Interestingly, salinity strengthens the relationship among bumblebees’ preference (L2), leaf nutritional (led by C, N, P, and S) and physiological (*Fv/Fm*) status, and vegetative and reproductive performance (Fig. 6D), rather than with flowers visited L1 + L2 (Fig. S6).

Moreover, bumblebees’ preference L2 was negatively correlated with leaf nutrients (Na, Mg, B, C/N) parameters (Fig. 6C) that negatively associated with plant growth and yield (Fig. S6). Among them, the salt-specific leaf Na concentration was the most negative trait associated with yield, as well as with bumblebees’ visits and preference (Figs. 6C, D; S6). Sulfur is another nutrient of interest, since leaf S was also one of the most positive traits associated with growth, yield, quality and pollinators’ preference under saline conditions (Fig. 6C, D), while this association was negative under control conditions (Figs. 6B; S5).

Overall, the results indicate that rootstock-mediated variation in bumblebees’ visits is associated with fruit yield and quality under both conditions. However, bumblebees appear to have a preference for the most healthy and productive graft combinations only under the selection pressure imposed by salt stress. Data also suggest that pollinators are more sensitive to the nutritional status of leaves than of flowers.

## Discussion

Increasing salt tolerance in horticultural crops through grafting requires the ad hoc development of specific rootstocks by screening, selection and breeding of graft-compatible germplasm, likely pyramiding several generic (vigor) and stress-specific traits related to local and (eco)systemic mechanisms. In this study, a biodiverse set of experimental rootstocks, including wild and domestic accessions, introgression lines, hormonal mutants, and intra-and interspecific hybrids, was grafted onto a single tomato cultivar and evaluated under commercial-like greenhouse conditions and moderate-to-high salinity. Rootstock biodiversity generated 2.4 and 2.7-fold variation in yield under optimal and saline conditions, respectively (Fig. 1), revealing valuable intra and inter genotypic variability in agronomic, physiological and agro-ecological traits, providing new insights into crop responses to salinity, and the identification of useful traits for breeding purposes. Particular emphasis is devoted to increasing knowledge of the environmental and physiological drivers influencing plant–bumblebee interactions and crop productivity.

Salinity reduced yield mainly by affecting vegetative growth, leaf area and PSII efficiency (*Fv/Fm*), in association with decreased leaf macro (C, N, P, K, Ca, S) and micro (Fe, Mn, B) nutrients, together with increased Na accumulation (Figs. 5G, J) and leaf C/N ratio (Fig. 5C). Interactions between mineral nutrients and C and N metabolism affect photosynthesis and protein biosynthesis (Sweetlove et al. 2010). Therefore, alterations in mineral status influenced C levels and the C/N balance, as supported by the strong relationship between *Fv/Fm* and these leaf nutritional parameters (Fig. S6). Salt-induced reduction in leaf nutrients, particularly N, P and S, contributes to less carbon assimilation under salinity (de Bang et al. 2021; Roșca et al. 2023), while carbon fixation is less affected than N assimilation, promoting accumulation of C-rich compounds and increasing the C/N ratio (Li et al. 2025). A high C/N ratio is a negative indicator of plant functioning and yield, which was intimately associated with other negative factors influencing growth, flower abortion, and yield, such as Mg and Na accumulation in leaves (Figs. 6C; S6). Given the tight coordination between C and N metabolism (Coruzzi and Zhou 2001; Nunes-Nesi et al. 2010; Gao et al. 2026), maintaining C/N homeostasis is considered a key adaptive strategy against adverse environmental conditions (Coruzzi and Bush 2001; Fañanás‐Pueyo et al. 2025; Li et al. 2025). Therefore, it is reasonable to expect a positive relationship between *Fv/Fm* and main leaf nutrients (C, N, P, K, Ca, S, Zn) under salinity, but a negative with a highly unbalanced C/N (Fig. S6). Together, those leaf nutritional and physiological factors reduced plant growth and the number and, especially, the weight of salinized fruits, as usually occurs under moderate-to-high salinity levels (Van Ieperen 1996; Cuartero and Fernández-Muñoz 1998). The reduction in the number of fruits was mainly explained by a reduction in the number of flowers linked to the reduced vegetative growth, since flower abortion and pollen viability were not significantly affected by the imposed salt stress (Fig. 2). However, the decrease in fruit weight can be explained by a lowered sink activity and the subsequent reduction in dry and fresh matter accumulation (Balibrea et al. 1996; Van Ieperen 1996; Albacete et al. 2014).

Interestingly, despite the nutritional stability in flowers under salinity (compared to control) to the detriment of leaves for most nutrients (Figs. 4–5; S4), yield-related traits in salinized plants were more strongly associated with leaf than flower nutritional status (Fig. 6C; S6). Indeed, none of the flower nutrients were positively associated with plant growth and yield under salinity (except Zn *vs* leaf area and weight), while Na, B, and, particularly, Ca were negative factors (Fig. 6D; S6). Moreover, Ca concentration in flowers was the most negative nutrient associated with plant physiology, growth and yield under salinity. Although Ca translocation to sink organs is generally limited (de Bang et al. 2021), stress-induced Ca waves from roots or leaves to flowers are considered systemic signals in response to injuries like heat or osmotic stresses (Grenzi et al. 2023; Bisht et al. 2025). Therefore, flower Ca concentration might be acting as a signal of stress sensitivity that is affecting yield. Interestingly, this putative salt-induced systemic signal seems to be mediated by the rootstock genotype. Curiously, although root fresh biomass positively influences plant growth and yield under optimal conditions, this parameter positively affected those yield-negative leaf traits under salinity, suggesting that root vigor per se could not be a beneficial rootstock trait for breeding against salinity, at least in the environmental conditions tested in this study.

These results indicate that nutritional status in leaves, rather than in flowers, is more important for sustaining plant growth and yield under salinity, probably through producing and transporting more organic assimilates to reproductive organs. Moreover, fruit quality (Brix) and yield, parameters usually negatively associated, showed a strong positive correlation under salinity (Fig. 6C), suggesting that rootstock-mediated performance under stress supports not only resources for generating vegetative and reproductive structures but also for filling them. The strong positive association between leaf C and N with growth, yield and fruit quality parameters supports this idea. Indeed, leaf C, N, P and S were negatively associated with flower abortion under salinity (Fig. S6). Therefore, rootstock-mediated securing mineral nutrients and balanced C/N in leaves, rather than in flowers, and restriction of toxic elements such as Na and B to the aerial part, are positive traits for improving tomato yield and quality under salinity.

Rootstock and/or salinity impact on flowers’ biology could have an effect on the pollination service by influencing the foraging decisions of the bumblebees. Indeed, pollinators’ preference at the variety-level has been reported in several crops (Klatt et al. 2013; Bergonzoli et al. 2022). In a previous study (Martínez-Andújar et al. 2023), bumblebee pollination was proposed as a rootstock-mediated trait in tomato influencing yield under nutrient deficiency (reduced NPK and P inputs), supported by the positive correlation between bumblebees’ preference (L2), yield and leaf nutritional (C, N, P, K, Zn) status. Similarly, salinity and the rootstock genotype generated physiological variation that correlated with changes in bumblebees’ foraging activity and preferences (L2) through nutritional and physiological interactions, which in turn aligned with yield. On average, salinity increased visitation rate (L1 + L2) but reduced pollinator preference (L2), probably because of an increase in smell signals and/or a reduction in the quality and quantity of food available in each flower. Importantly, while the percentage of flowers visited (L1 + L2) was related to yield under whatever conditions (Martínez-Andújar et al. 2023; Figs. 3C, E), the relationship between bumblebees’ preference (long visits, L2) and yield was only evident under environmental pressure imposed by the nutrient deficiency (Martínez-Andújar et al. 2023) or salinity, rather than under optimal conditions (Martínez-Andújar et al. 2023; Figs. 3D, F; 6A, C). Notably, the traits most strongly associated with yield under salinity (Fig. 6C), including leaf Na concentration, K:Na ratio, Fv/Fm, and leaf C, N, P and S status, were also associated with bumblebee preference (Fig. 6D), revealing a substantial overlap between the physiological determinants of plant productivity and pollinator foraging decisions under stress. Indeed, the relationship between *Fv/Fm*, C/N ratio, leaf mineral nutrients and bumblebees’ preference L2 under salinity was absent under control conditions (Figs. 6, S5, S6). Moreover, pollinating activity (L1 + L2) and preference (L2) were more associated with leaf than with flower nutritional traits, which was reinforced by salinity, suggesting that leaf physiological status (source activity) is reflected in the strength of the mutualistic interaction. Salinity decreased C and N concentrations and increased C/N ratio in leaves, without affecting the flowers (Fig. 5). Regardless of the impact of the stress on those major nutrients and their balance in leaves, C and N seem to influence bumblebees’ preference. Leaf C was consistently associated with L2 when it was reduced by the stress (this study) or when it was accumulated under P and NPK deficiencies (Martínez-Andújar et al. 2023), but not under optimal conditions (both studies), thus coupling photosynthesis, carbon metabolism, pollinator activity and plant performance under environmental pressure. Among nutrients, leaf S was also one of the most positive traits associated with growth, yield, quality and bumblebees’ activity under saline conditions. Sulphur volatiles are determinant of tomato aroma and influence honeybees, butterflies and bumblebees foraging activity (Haneklaus et al. 2005; Bloem et al. 2010; Shuttleworth and Johnson 2010). Moreover, heterografts increased bumblebees’ activity along with the S-containing amino acid Met in tomato fruits (Ormazabal et al. 2024). Therefore, pollinators’ foraging decisions may provide non-invasive approach to phenotype the integration of the reproductive development and carbon metabolism across environments for breeding decisions, although further validation across diverse environmental conditions and genetic backgrounds will be required before practical implementation in breeding programmers.

Finally, it is well-known that Na accumulation and the K/Na homeostasis in leaves are major negative and positive factors affecting plant physiology and productivity under salt stress, respectively. In the present study, leaf Na concentration and K/Na ratio exhibited the largest rootstock-dependent variation across the population, and a significant genotype effect was detected for both traits, highlighting ion homeostasis as a major target of rootstock-mediated adaptation to salinity. While leaf K has been previously linked to bumblebees’ preference under low-P and NPK (Martínez-Andújar et al. 2023), a novel finding is that rootstock-mediated variation in leaf Na showed the strongest negative association with pollinators’ preference L2. Indeed, although Na in nectar can attract pollinators (Finkelstein et al. 2022; Lovett and Carr 2023), Na does not accumulate in pollen grains (tomato does not produce nectar) of tomato flowers under salt stress (Ghanem et al. 2009), and bulk Na concentration in flower structures was not associated with L2 in this study. Since the negative effect of Na on leaves negatively affected other nutritional (C, N, P, K, Ca, S, Fe, Zn) and physiological (*Fv/Fm*) traits, the results suggest that bumblebees may be responding to leaf physiological status, potentially mediated by source–sink relationship with flowers. Indeed, albeit flower abortion was not significantly affected by salinity in the whole population (Fig. 2C), this trait was positively correlated with N in flowers and C/N in leaves, but negatively with C and N in leaves. Moreover, since C and, to a higher extent N, were reduced by salinity in leaves but not in flowers, the results suggest that N concentration and C/N balance in leaves protect flower abortion rather than in flowers, probably through maintaining proper and balanced primary metabolism in leaves and C/N-assimilate transport to flowers. Following uptake and translocation from roots to leaves, inorganic N is assimilated into amino acids and re-distributed within the plant from source to sink tissues. Overall, this optimized leaf functioning under salinity seems to be reflected in pollinators foraging decisions, as supported by the positive correlation of L2 with yield, biomass, pollen viability, fruit number and weight, leaf nutrient (N, P, K, Ca and S) concentrations and chlorophyll fluorescence (*Fv/Fm*). Those results suggest a potential role for pollinators as natural phenotypers of plant physiological status under environmental constraints (Pérez-Alfocea et al. 2024), similarly to how they act as ecological drivers of adaptive evolution in nature (Dorey and Schiestl 2024).

## Conclusions

The main impact of rootstocks on the scion performance under salinity would be expected to occur through root vigor (RFW), as observed under control conditions. However, in this study, the root biomass did not correlate with plant growth and yield under salinity, while RFW negatively (N, P, K, S) and positively (Na, B) influenced leaf nutrient concentrations, suggesting that other local (e.g., root architecture) or systemic (root-to-shoot communication) factors are positively influencing growth and yield under salinity through the capture and distribution of essential macronutrients and toxic elements. Thus, rootstock-mediated Ca accumulation in flowers, in detriment of leaves, seems to act as a sensitive signal of saline stress negatively influencing yield. The interaction between the major rootstock-mediated variable leaf Na concentration and major leaf nutrient status and the subsequent impact on the photosynthetic apparatus and unbalanced C/N metabolism represent key factors strongly associated with plant growth, yield reduction and bumblebees’ preferences. The association between rootstock-mediated agronomic (plant growth and yield), physiological (*Fv/Fm*, leaf nutrient status), and ecosystemic (pollinators’ preference) traits was evident under saline stress, but not under control conditions. Collectively, these results support a conceptual framework in which rootstock-mediated ion homeostasis—particularly leaf Na concentration and the K:Na ratio—and nutrient balance shape plant physiological status under salinity, as reflected by the photosynthetic performance and C/N metabolism, which in turn influences both bumblebee foraging decisions and crop productivity. Moreover, the leaf rather than the flower traits analyzed appears to be more influential for such associations, reinforcing the role of leaf metabolism and functioning in mediating mutualistic interactions perceived by bumblebees through the flowers. The conventional indirect observational method used for monitoring pollination activity based on bite marks on anthers provides a practical proxy for pollinator activity; it is limited by the subjective observations of static traits over time in a high number of plants, and by the intensive labor requirements (Rhodes et al. 2022). In this context, a new RFID-based method for automated sequential analysis of complex genotype × environment × pollinator interactions at phenotyping scale has now been developed (Jiménez et al. 2026). This technological breakthrough will contribute to further study and validating the relationship between plant resilience, pollinator foraging decisions and leaf and flower C/N metabolism. However, further validation across additional environmental scenarios, crop genetic backgrounds and growing conditions will be required before pollinator-assisted phenotyping can be routinely implemented in breeding programs.

## Supplementary Information

Below is the link to the electronic supplementary material.

Supplementary file1 (PDF 6970 KB)

## Data Availability

The data that support the findings of this study are available from the corresponding author upon reasonable request.
