# Supplementary material for: Salinity strengthens plant–pollinator mutualism through leaf physiological status in grafted tomatoes
Source: Planta. 2026 Aug 18;264(4):100. doi: 10.1007/s00425-026-05110-y (PMC13486138; doi:10.1007/s00425-026-05110-y)
Supplement: Supplementary file 1 [file 425_2026_5110_MOESM1_ESM.pdf]

Electronic supplementary material

Prudencio *et al*.

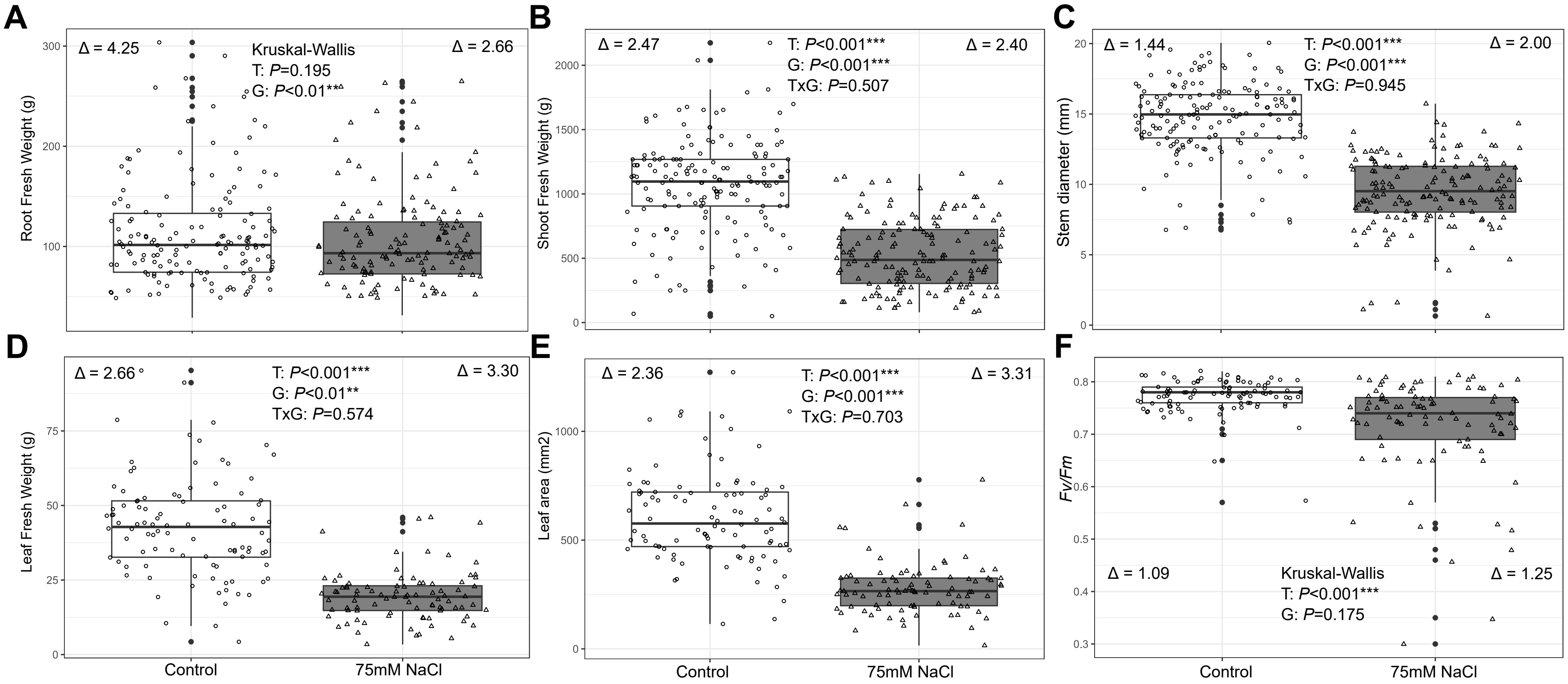

Fig.S1 Boxplot with individual data points showing the root and shoot fresh weight and stem diameter (A-C *n*= 156/157 for control and *n*=146/150 for NaCl conditions) and leaf fresh weight; leaf area and *Fv/Fm* (D-F n=98 for control and *n*= 88/92 for NaCl conditions) of *Solanum lycopersicum* cv. 'Unidarkwin' plants grafted onto different rootstocks growing under control and NaCl conditions during 78 days. Δ indicates fold change in each case between the highest and lowest rootstock value, on average. Significant effects on treatment (T), genotype (G), and their interaction (T × G) tested by ANOVA are indicated. Otherwise, differences between T and G were tested by Kruskal-Wallis test. Significance is indicated by asterisks (*=*P*<0.05, **=*P*<0.01, ***=*P*<0.001).

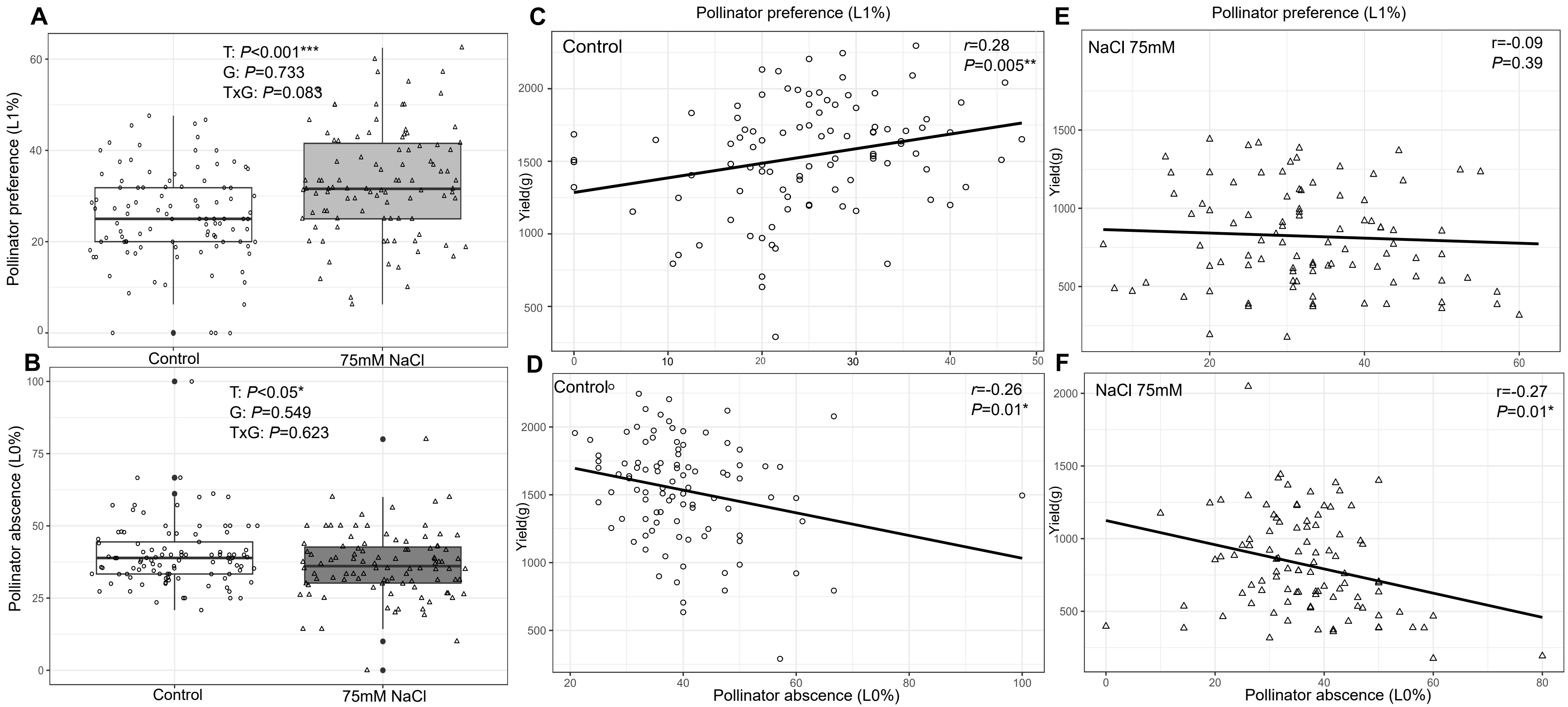

Fig.S2 Scatterplot with individual data points showing percentage of flowers with short visits (L1, A) or percentage of flowers non-visited (L0, B) by the treatment (*n*=101 for control and 98 for NaCl treatment). Significant effects of treatment (T), genotype (G), and their interaction (T × G) are indicated (*=*P*<0.05) according to ANOVA test. Spearman correlations between pollinator activity or absence to yield under control (C, D) and NaCl (E, F) conditions, with correlation coefficient (*r*) values and *P*-values indicated at the top right of each panel.

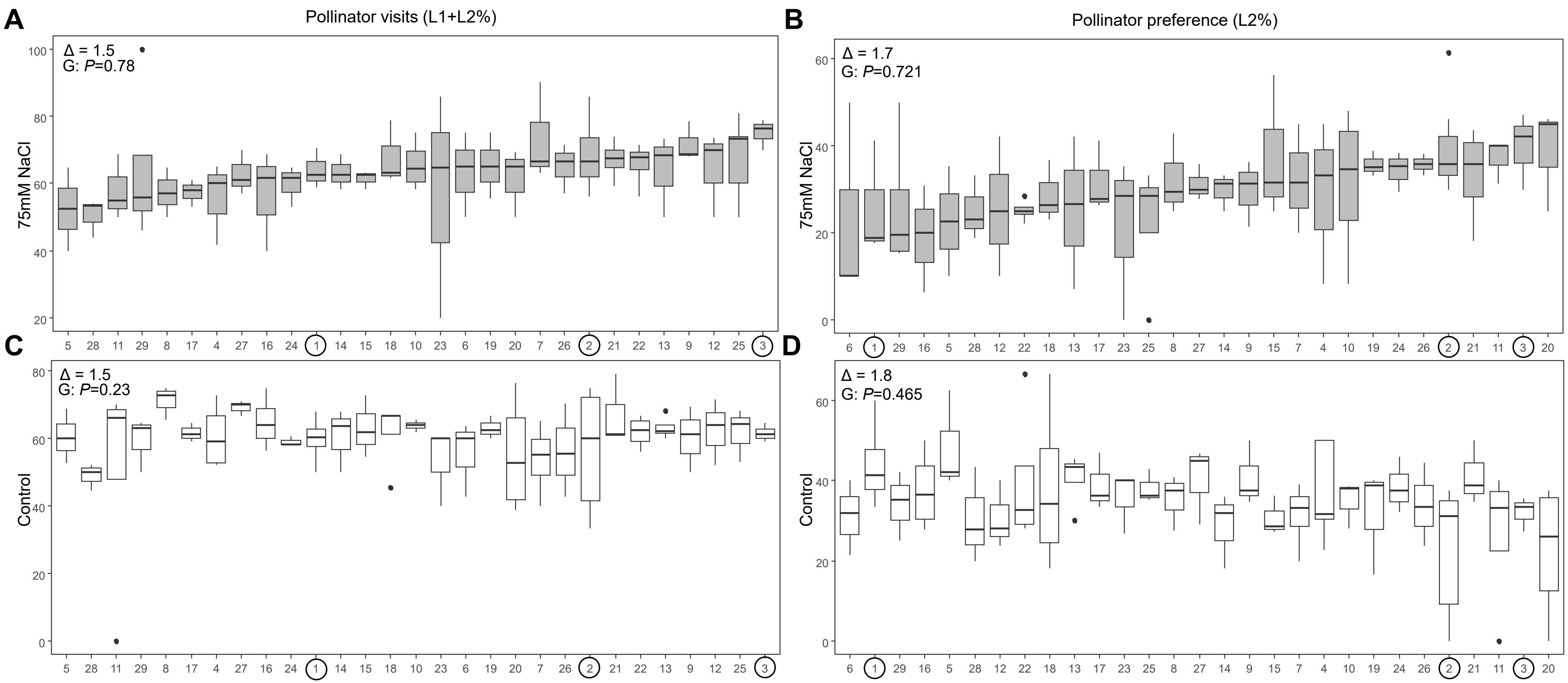

Fig.S3 Boxplot showing the pollination (L1+L2) and pollinator's preference (L2) of *Solanum lycopersicum* cv. 'Unidarkwin' grafted onto different rootstocks growing under 75mM NaCl (A, B) and control (C, D) conditions during 100 days. *n*=3-6, except for non-grafted control under control (2, *n*=7) and for the self-grafted under control (3, *n*=9) and NaCl (*n*=11) conditions. Non-Grafted (NG) and Self-Grafted (SG) controls correspond to number #1 and #2, and commercial rootstock 'Maxifort' corresponds to genotype number #3 (circled). ANOVA *P*-value between genotypes is indicated in each case.

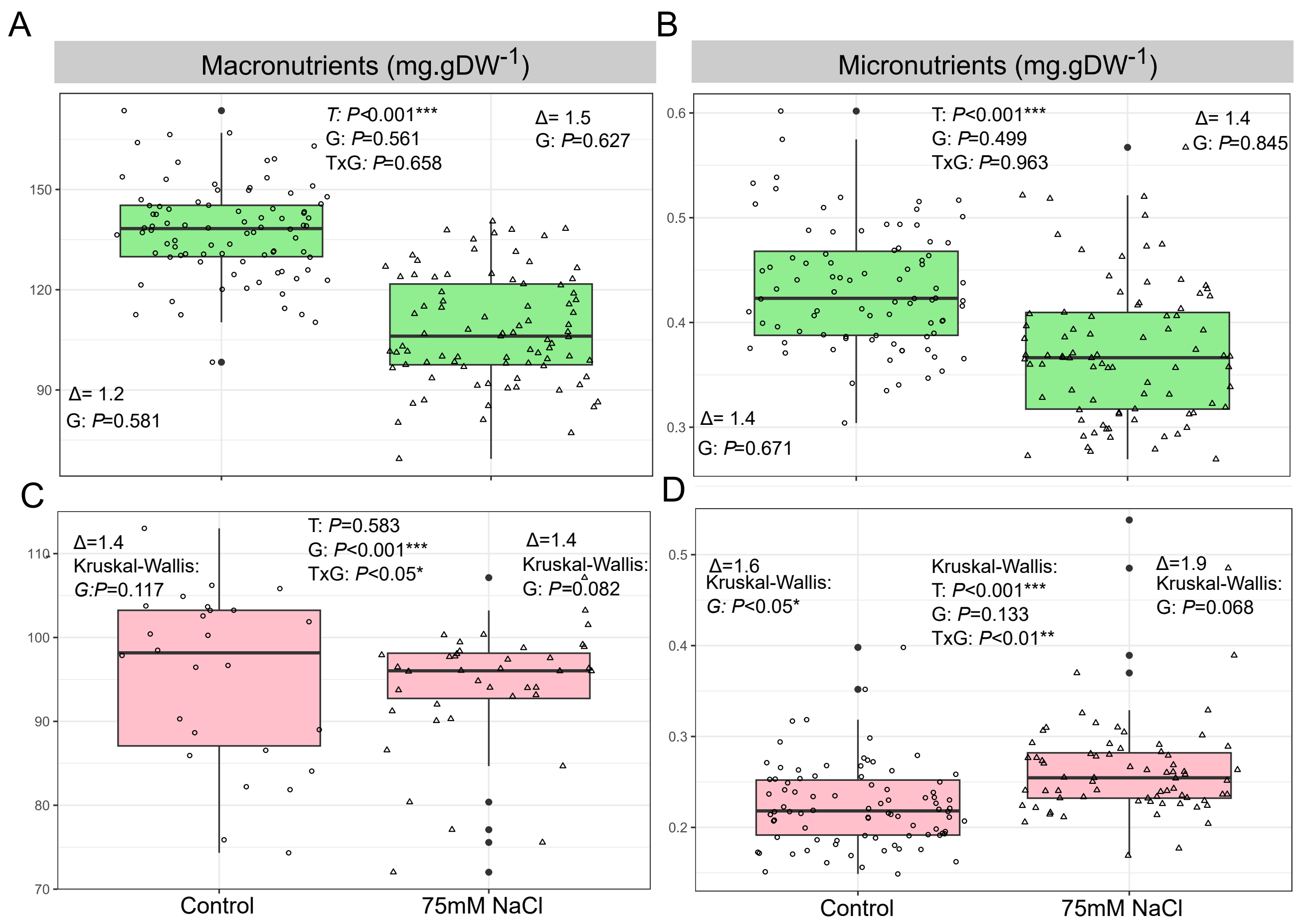

Fig.S4 Boxplot with individual data points showing the macronutrients (N, P, K, Mg, S and Ca) and micronutrients (N, P, K, Mg, S and Ca) from leaves (A and B) and flowers (C and D) of *Solanum lycopersicum* cv. 'Unidarkwin' plants grafted onto different rootstocks growing under control and 75mM NaCl conditions during 95 days. Δ indicates fold change in each case between the highest and lowest rootstock graft, on average. Significant effects of treatment (T), genotype (G), and their interaction (T × G) tested by ANOVA or Kruskal-Wallis are indicated.

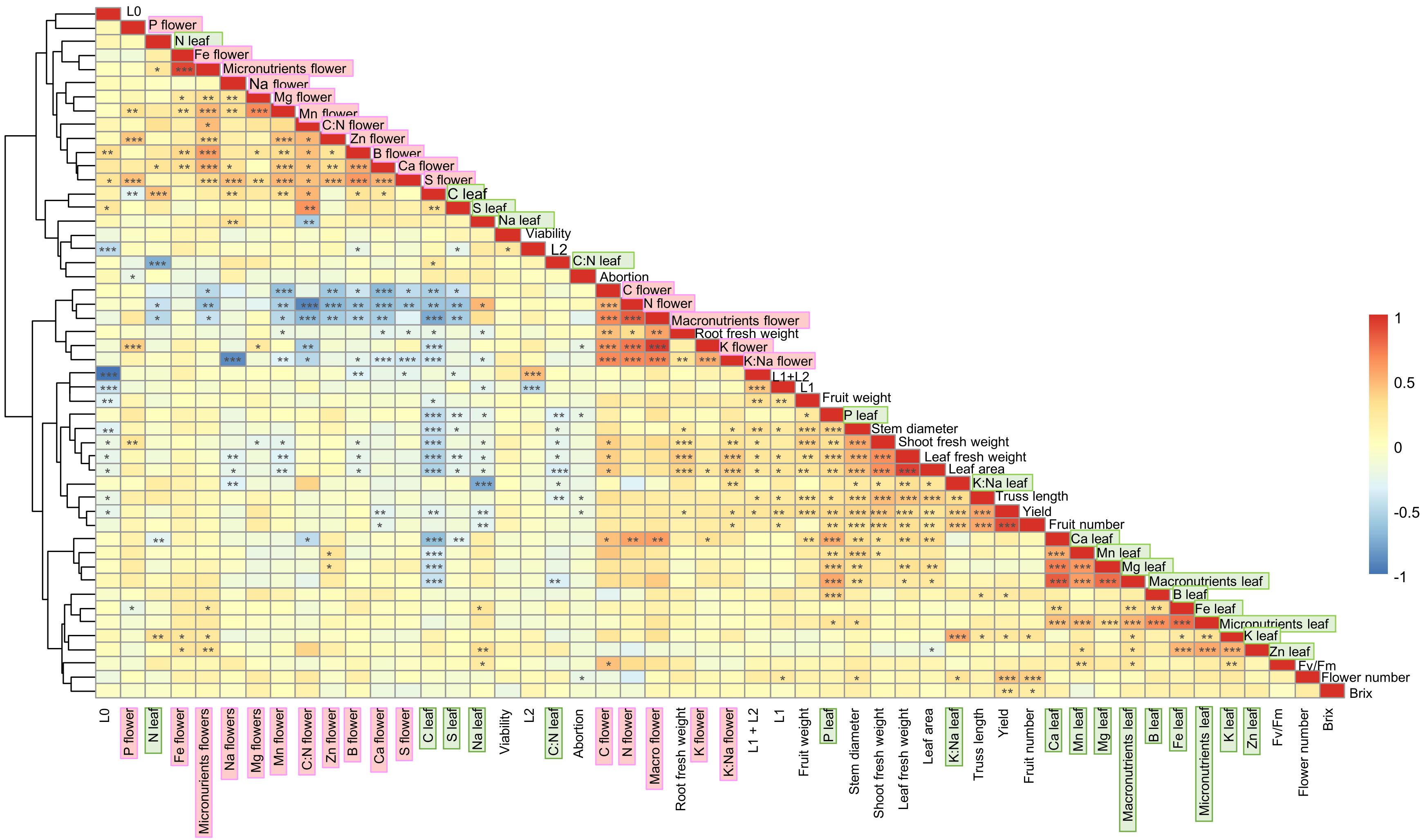

Fig.S5 Heatmap showing Spearman correlations between leaf and floral macro- (Ca, Na, K, P, S, Mg) and micronutrients (Fe, Zn, B and Mn) and yield parameters (yield, fruit number, fruit truss length), growth parameters (leaf fresh weight, leaf area, stem diameter, shoot fresh weight and root fresh weight), pollinator activity level (percentage of L0, L1, L2, L1+L2), chlorophyll fluorescence (*Fv/Fm*), brix values, flower abortion and pollen viability under control conditions. Leaf (green) and flower (pink) nutrients are included in boxes. Level of significance is indicated by asterisks (*=P<0.05, **=P<0.01 and ***=P<0.001).

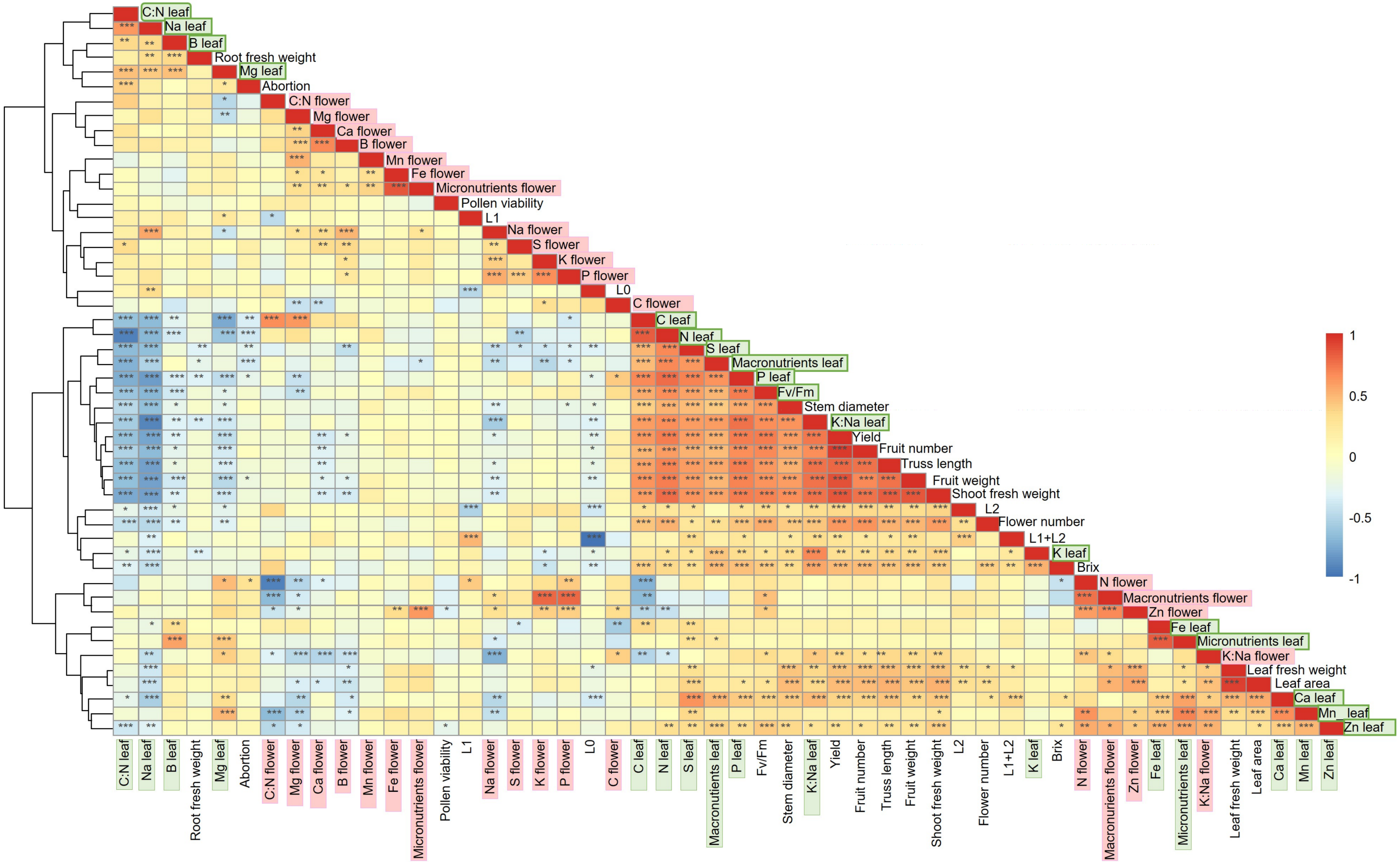

Fig.S6 Heatmap showing Spearman correlations between leaf and floral macro- (Ca, Na, K, P, S, Mg) and micronutrients (Fe, Zn, B and Mn) and yield parameters (yield, fruit number, fruit truss length), growth parameters (leaf fresh weight, leaf area, stem diameter, shoot fresh weight and root fresh weight), pollinator activity level (percentage of L0, L1, L2, L1+L2), chlorophyll fluorescence (*Fv/Fm*), brix values, flower abortion and pollen viability under salt stress conditions. Leaf (green) and flower (pink) nutrients are included in boxes. Level of significance is indicated by asterisks (*=P<0.05, **=P<0.01 and ***=P<0.001).
